# Cooperative Behavior in the Ultimatum Game and Prisoner’s Dilemma Depends on Players’ Contributions

**DOI:** 10.3389/fpsyg.2017.01017

**Published:** 2017-06-16

**Authors:** Amy R. Bland, Jonathan P. Roiser, Mitul A. Mehta, Thea Schei, Barbara J. Sahakian, Trevor W. Robbins, Rebecca Elliott

**Affiliations:** ^1^Neuroscience and Psychiatry Unit, University of ManchesterManchester, United Kingdom; ^2^Institute of Cognitive Neuroscience, University College LondonLondon, United Kingdom; ^3^Institute of Psychiatry, Psychology and Neuroscience, King’s College LondonLondon, United Kingdom; ^4^Department of Psychiatry, University of CambridgeCambridge, United Kingdom; ^5^Department of Psychology, University of CambridgeCambridge, United Kingdom; ^6^Behavioural and Clinical Neuroscience Institute, University of CambridgeCambridge, United Kingdom

**Keywords:** Ultimatum Game, Prisoner’s Dilemma, fairness, cooperation, contribution, sex differences

## Abstract

Economic games such as the Ultimatum Game (UG) and Prisoner’s Dilemma (PD) are widely used paradigms for studying fairness and cooperation. Monetary versions of these games involve two players splitting an arbitrary sum of money. In real life, however, people’s propensity to engage in cooperative behavior depends on their effort and contribution; factors that are well known to affect perceptions of fairness. We therefore sought to explore the impact of relative monetary contributions by players in the UG and PD. Adapted computerized UG and PD games, in which relative contributions from each player were manipulated, were administered to 200 participants aged 18–50 years old (50% female). We found that players’ contribution had large effects on cooperative behavior. Specifically, cooperation was greater amongst participants when their opponent had contributed more to joint earnings. This was manifested as higher acceptance rates and higher offers in the UG; and fewer defects in the PD compared to when the participant contributed more. Interestingly, equal contributions elicited the greatest sensitivity to fairness in the UG, and least frequent defection in the PD. Acceptance rates correlated positively with anxiety and sex differences were found in defection behavior. This study highlights the feasibility of computerized games to assess cooperative behavior and the importance of considering cooperation within the context of effortful contribution.

## Introduction

Economic games, such as the Ultimatum Game (UG) and Prisoner’s Dilemma (PD) have become popular paradigms for exploring social decision-making. Indeed, the robust behavioral patterns observed across studies suggest that the UG and PD may provide important assessment tools for evaluating social cognition.

The UG, developed by Güth et al. (1982), involves two players who are asked to divide a given amount of money (e.g., £10). The proposer must decide how the money should be divided, whilst the receiver may choose to accept or reject the offer (e.g., the proposer could choose to keep £8 and offer the receiver £2). If the receiver accepts the offer, both players receive the agreed amount, but if the receiver rejects, neither receive anything. The striking finding observed in the UG is that players do not behave according to predictions made by classical economic theories of utility (Von Neumann and Morgenstern, 1947). Specifically, these theories assume that the receiver should accept any offer proposed in order to maximize their own financial gain since the alternative is to receive nothing. Instead, however, players show a consistent willingness to forfeit gains by rejecting offers that are deemed to be unfair. Previous studies suggest that at least half of receivers reject offers below 30% (Güth et al., 1982; Rubinstein, 1982; Thaler, 1988), a finding that has been replicated consistently, with some variations observed in gender (Eckel and Grossman, 2001), social distance (Page et al., 2000), hypothetical vs. real money rewards (Fantino et al., 2007; Gillis and Hettler, 2007) and cultures (Henrich et al., 2005). Acceptance rates can differ quite dramatically between societies, therefore any generalizing from behavior in the games to potential social cognition deficits may only be applicable within a particular culture.

The UG is typically played only once with each ‘opponent’ so that reciprocation cannot explain this robust behavioral pattern. Rejection of unfair offers has therefore been attributed to individuals’ preferences for fairness (Bolton, 1991), a desire to punish socially unacceptable behavior (Fehr and Schmidt, 1999) or an important adaptive mechanism which serves to maintain social reputation and group cooperation (Nowak et al., 2000). These accounts are not mutually exclusive.

In the PD, (Poundstone, 1992), two players can choose to either cooperate or defect in a social exchange situation. For example, in a monetary version of the PD, two players are asked independently whether they would like to cooperate or defect a given amount of money (e.g., £10). If both players choose to *cooperate*, the money is divided equally between them (£5 each). However, if player A chooses to *cooperate* whilst player B chooses to *defect* then player B receives all of the money and player A receives nothing. If both players choose to defect then neither receives any money. Therefore this game highlights the conflict between pure self-interest and mutual cooperation (Axelrod and Hamilton, 1981). Similarly, the Dictator Game (DG) allows a proposer or “dictator” to determine how to split a payoff with the receiver (Kahneman et al., 1986; Forsythe et al., 1994). However, unlike the UG, the recipient is passive and does not have a choice whether to accept or reject the offer. The iterated PD, which involves playing the same player repeatedly, also allows individuals to build strategic relationships by reacting to and punishing an opponent’s past behavior. For instance, players can use a tit-for-tat strategy whereby a player will respond to their opponent with, however, that opponent treated them most recently. Players form a reciprocating strategy where mutual cooperation in the iterated PD is usually higher than in the single shot PD (Axelrod and Hamilton, 1981).

Ultimatum Game and PD paradigms typically use a predetermined and often arbitrary sum of money, to which players have made no contribution. However, in real life settings, rewards are rarely obtained with little or no exertion. Usually, rewards are achieved through effort and are differentially satisfying depending on the relative effort applied and the relative rewards gained by others (Tabibnia and Lieberman, 2007). For example, consider that two colleagues complete a project and are rewarded with a share of the team bonus. If the bonus is evenly distributed between the colleagues, according to traditional UG findings, this would be considered a fair split and both would be satisfied with their equal share of the bonus. However, if one colleague had completed a larger share of the work whilst the other had exerted minimal effort, a 50/50 split might not be deemed fair, especially by the employee who contributed the most to the project. This more sophisticated assessment of fairness may be more typical of many real life social scenarios than the traditional UG and PD games. Indeed, it has been argued that participants in a typical UG may agree on an equal split simply because the stake is a “free gift” from the experimenter (Berger et al., 2012). Therefore taking into account the relative effort in relation to the reward obtained may have important implications for understanding cooperative behavior.

Recently, several investigators using social exchange paradigms have tried to move away from bargaining over a “free gift” from the experimenter. One approach is to delay the reward, with participants having to decide how to split the waiting time between them (Berger et al., 2012). In other studies, players had to decide how to share a “workload” such as solving mathematical questions (Ciampaglia et al., 2014). Studies have shown that individual contributions create “entitlements” which play an important role in offers proposed. For example, in the DG when people earn the right to take the role of dictator, they give less (Hoffman et al., 1994). Similarly dictators give less when they earn income themselves (in an academic test) compared to when income is determined by the experimenter (Cherry et al., 2002; Oxoby and Spraggon, 2008) or won on a coin toss (Ruffle, 1998). Carr and Mellizo (2013) reported an adapted UG where responders solve math problems to generate an endowment whereby responder-produced endowments increase offers while acceptance rates are higher suggesting that cooperative behavior is driven by the source of the entitlement. Further Cappelen et al. (2013) conducted a real-effort DG where students in two of the world’s richest countries, Norway and Germany, were matched directly with students in two of the world’s poorest countries, Uganda and Tanzania who generated money through a word entry task and found that participants assigned greater importance to individual contributions in their distributive choices than they did needs and nationality. Individual contributions, and thus entitlement therefore, appear to play a vital role in people’s distributive behavior (Hoffman et al., 1994; Konow, 1996, 2010; Cherry et al., 2002; Frohlich et al., 2004; Gächter and Riedl, 2006; Cappelen et al., 2007, 2010; Oxoby and Spraggon, 2008).

However, a major drawback of the UG and PD is that they can require a long and complex set-up with multiple players or confederates, which involves pairing participants either in person or more recently via the internet. In clinical trials and studies focusing on patient populations, this may not be possible given the logistics involved. Some studies match each player’s individual decision with previous real player responses. However, data can only be obtained in response to a previous player’s individual behavior, which may be aggressive or cooperative therefore not allowing each player to play under the same circumstances. Playing against a computer, however, allows the systematic manipulation of an opponent’s behavior to enable investigation of player behavior against different types of opponents. This allows the assessment of punishment of uncooperative behavior and negative reciprocity, (e.g., Fehr and Schmidt, 2006) in response to opponent defection. We sought to explore the effects of contribution and entitlement on reciprocity using an iterated PD so that we could establish whether participants who had contributed more to the pot of money were more likely to defect compared to when the opponent contributed more and when contributions were equal when an opponent has been aggressive or cooperative. We therefore aimed to develop versions of these economic tasks that are short, one player and easy to administer on a laptop or tablet, either in a laboratory setting or at home. Participants were not deceived in any way to believe their avatar opponent was a real person, nor were they led to believe that the responses of the avatar were based on previous participants’ responses. To our knowledge, there has been no empirical exploration of the impact of relative monetary contributions and entitlement in the UG and PD using computer avatar opponents.

The aim of this study was to evaluate how relative contribution to the common resource, and therefore a sense of entitlement, affects cooperative behavior. We predicted that varying levels of contribution would shift the distribution of offers, such that greater contribution would result in significantly less cooperative behavior. We predicted that this would be manifested in more rejection of offers in the UG and more defecting behavior in the PD.

## Materials and Methods

### Participants

Adapted UG and PD tasks were administered to two hundred healthy volunteers. Half of participants were degree educated and recruited from the Universities of Manchester and Cambridge and the other half of participants were non-degree educated from the wider Greater Manchester and Cambridgeshire areas. Participants were not recruited if they had previously participated in similar cognitive tasks within the departments. Participants were included if they met the following criteria: 18–50 years old; no previous or current psychiatric disorders; no first degree relatives suffering from any psychiatric disorders; smoking less than five cigarettes per day; drinking less than the United Kingdom government guidelines for weekly alcohol intake (at the time of the study – males: 3–4 units per day; females: 2–3 units per day) and fluent in English. Participants completed the Brief Symptom Inventory (Derogatis and Melisaratos, 1983), meeting the criteria for Adult Non-patients. Participants were further interviewed using the Mini International Neuropsychiatric Interview (Sheehan et al., 1998) and were excluded if they met the criteria for any psychiatric diagnosis.

Participants’ mean age was 26.77 years (*SD* = 9.81), with a mean educational level of 14.40 years (*SD* = 2.01) and a mean WTAR score was 112.18 (*SD* = 6.29). The sample consisted of 100 male and 100 female participants, half of whom were educated to degree level. The overall ethnic/racial distribution of the sample was 78.5% White (*N* = 157), 3.5% Afro Caribbean (*N* = 7), 5% Asian-Indian (*N* = 10), 4.5% East-Asian (*N* = 9), 4.5% Mixed (*N* = 9) and 4% other (*N* = 8).

### Procedure

The UG and PD were administered as part of the EMOTICOM neuropsychological test battery (Bland et al., 2016). This study was approved by the University of Manchester and the University of Cambridge Research Ethics Committees and participants provided written informed consent after the study procedures were explained. Participants were reimbursed for their time and travel expenses. Participants completed the task on a touchscreen laptop (Dell XT3) using PsychoPy software (Peirce, 2007) in a quiet testing room. Participants’ IQ was estimated using the Wechsler Test of Adult Reading (Wechsler, 2008) and they completed two current mood state questionnaires, the Profile of Mood States (POMS: Shacham, 1983) and the State subscale of the State-Trait Anxiety Inventory (STAI: Spielberger et al., 1970). Prior to the visit, participants completed the following questionnaires online: Big Five Personality Inventory (John et al., 1991), Eysenck Personality Inventory (EPQ: Eysenck and Eysenck, 1991), the Barratt Impulsivity Scale (BIS-11: Patton et al., 1995), the UPPS-P Impulsive Behavior Scale (Whiteside and Lynam, 2003) and the Trait subscale of the STAI (Spielberger et al., 1970).

### Task Design

The presentation of the UG and PD was randomized across participants.

#### Ultimatum Game (UG)

**FIGURE 1:**
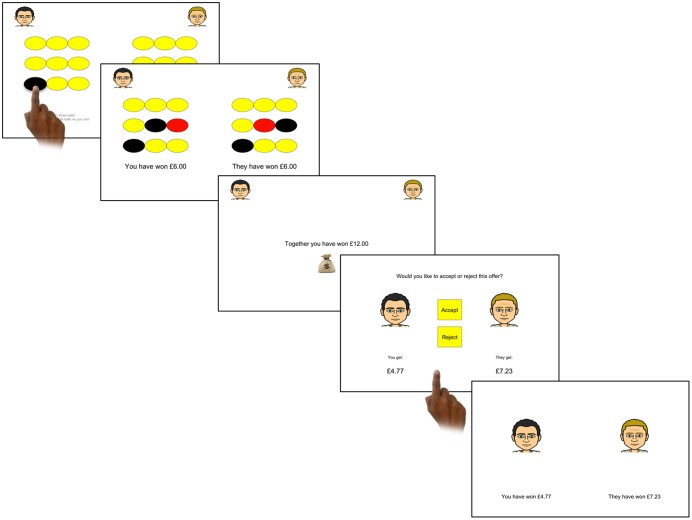
One trial of the Ultimatum Game. First, participants had to select three ovals before receiving feedback of how much they and their computer opponent have contributed. A screen then showed the total of their combined earnings. In this trial the computer decided how the money should be split and the participant chose whether to accept or reject the offer. If the participant accepted, the earnings were distributed accordingly; however, if the participant rejected, neither player received anything. Finally participants were shown feedback of how much they had received.

In the adapted UG, participants first chose an avatar to represent them in the game. On each trial participants were paired with a random opponent avatar and were required to complete a task in which they could each earn money to contribute to a stake (see **Figure 1**). Specifically, nine yellow ovals were presented on the screen and participants had to select three to uncover. If the oval turned black, participants earned £3 whereas if the oval turned red, participants received nothing. Therefore the value that a participant could contribute on any given trial ranged from £3–£9. Trials were manipulated so that one of three outcomes occurred: (1) the participant contributed more; (2) the opponent contributed more; (3) both players contributed equally. Participants contributed all their earnings and this money was then combined with their opponent’s earnings (also ranging from £3–£9). The total share (from both parties) was the same in the different player-contribution conditions.

Next, participants were informed who would decide how the money would be split. Participants played against 51 players; on 70% of the trials the computer opponent decided how to split the money similarly to the classic UG, whereas on the remaining 30% the participant decided how to split the money. If the opponent divided the money, the participant had the choice to either accept or reject their offer. These offers had seven levels where the participant was offered 10, 20, 25, 30, 35, 40, or 50%. If the participant accepted the offer, both players received the allotted amount; if they rejected the offer, neither received anything. When the participant was allowed to choose how to split the money, they could choose to offer their opponent 20, 30, 40, or 50%. The computer opponent was programmed to always accept the offer although this was not made explicit to the participant.

Importantly, evidence suggests that UG behavior differs depending on whether the stake is hypothetical or real, with participants in hypothetical situations offering less (Fantino et al., 2007; Gillis and Hettler, 2007). In this adapted UG, we therefore opted to use real rewards and thus paid participants according to their trial-averaged earnings (*total amount accepted* + *share of total proposed/number of trials).*

#### Prisoner’s Dilemma (PD)

Similar to the UG, participants were first required to choose an avatar to represent them in the game. Participants then played nine games of an iterated PD against three opponents (27 rounds in total). The first opponent adopted a suspicious “tit for tat” strategy which involved the opponent defecting on the first trial and subsequently retaliating to the participants’ choices, i.e., if the participant had split on the previous trial, then the opponent would split on the current trial; whereas if the participant had stolen on the previous trial, the opponent would defect on the current trial. The second opponent adopted a “tit for two tats” strategy which meant that participants would have to defect on two consecutive trials for the opponent to retaliate. The final opponent was cooperative and always split regardless of the participant’s behavior. These opponent strategies were not made explicit to the participant and each participant received the same order of opponents from suspicious to cooperative. The task was designed in this way in order to avoid participants beginning with a cooperative player and thus cooperating all the way through the task. Unlike the UG, a one-shot game was not used and instead an iterated PD was utilized in order to explore reciprocity.

**FIGURE 2:**
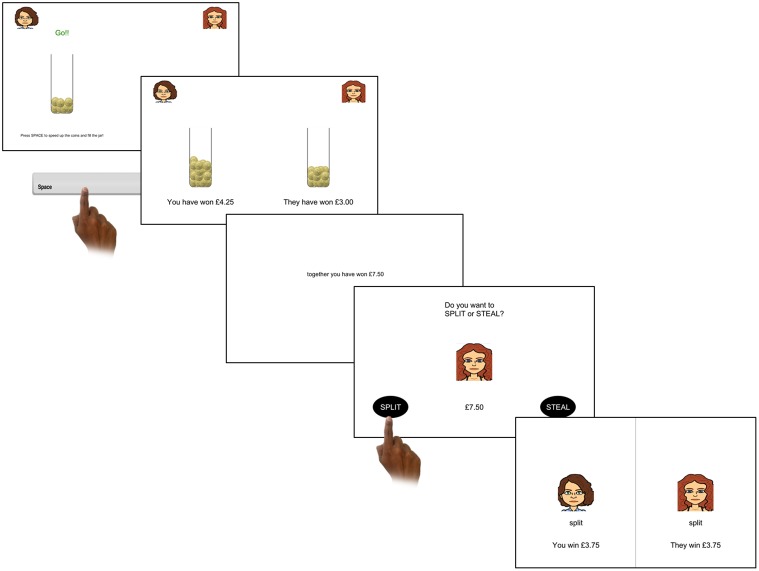
One trial of the Prisoner’s Dilemma. First, participants repeatedly pressed the spacebar as fast as possible; the more presses they completed the quicker the jar filled with coins. Participants then received feedback showing how much they and their computer opponent had contributed, and a subsequent screen showed the total combined earnings. Then participants made the decision to split or defect this combined pot. In this trial the participant and the computer both decided to split and so the earnings were split evenly.

On each trial, participants were initially required to complete a task in which they could earn money through exerting effort to contribute to a stake (see **Figure 2**). This involved pressing a button as fast as possible to fill a jar with coins. The faster they pressed the button the more the jar would fill. Similarly to the UG, this money was then combined with their opponent’s earnings. This was manipulated so that one of three outcomes occurred: (1) the participant contributed more; (2) the opponent contributed more; (3) both players contributed equally. The total share (from both parties) was different in the different player-contribution conditions [i.e., the number of coins the opponent earned was either increased above the players amount in the condition where opponents contributes more (130% of players contribution) or decreased below the player contribution where the player contributes more (70% of players contribution)]. Next, participants were given the option to cooperate “split” or defect “steal.” If both the participant and the opponent split the money, each received 50% of the pot; whereas if both chose to defect, neither received any money. If the participant chose to defect and the opponent chose to split then the participant would receive the full amount and the opponent received nothing; whereas if the participant chose to split but the opponent chose to defect then the opponent would receive the full amount and the participant received nothing. This payoff matrix was used in order to better match the possible payoffs in the UG.

### Analysis

All statistics were computed with SPSS statistical software (IBM SPSS Statistics Version 20.0). For the UG, receiver data were entered into a 3 (contribution: participant contributed more, opponent contributed more, equal contributions) × 7 (avatar offer: 10, 20, 25, 30, 35, 40, and 50%) repeated measures analysis of variance (ANOVA). Proposer data were entered into a 3 (contribution: participant contributed more, opponent contributed more, equal contributions) × 4 (participant offer: 50, 40, 30, and 20%) repeated measures ANOVA. For the PD, data were entered into a 3 (contribution: participant contributed more, opponent contributed more, equal contributions) × 3 (avatar strategy: suspicious, tit for two tats, cooperative) repeated measures ANOVA. For *post hoc* analyses, contrasts of simple main effects were conducted using paired samples *t*-tests. Pearson’s *r* was used to correlate behavior within and between tasks, and with personality and mood questionnaires. Statistical correction for multiple comparisons was applied to the latter correlations, due to the high number of relationships tested (*0.05/n; n* was determined as 24, i.e., 6 questionnaires × 4 task variables). On all tests *p* < 0.05 was considered significant and 0.05 < *p* < 0.1 a trend toward significance. With 200 participants we had 90% power to detect differences between conditions of *d* = 0.23 at *P* = 0.05 (two-tailed), and correlations with mood and personality variables of *r* = 0.30 at *P* = 0.002 (two-tailed, corrected for 24 comparisons).

## Results

### Ultimatum Game

#### Offers Accepted

**FIGURE 3:**
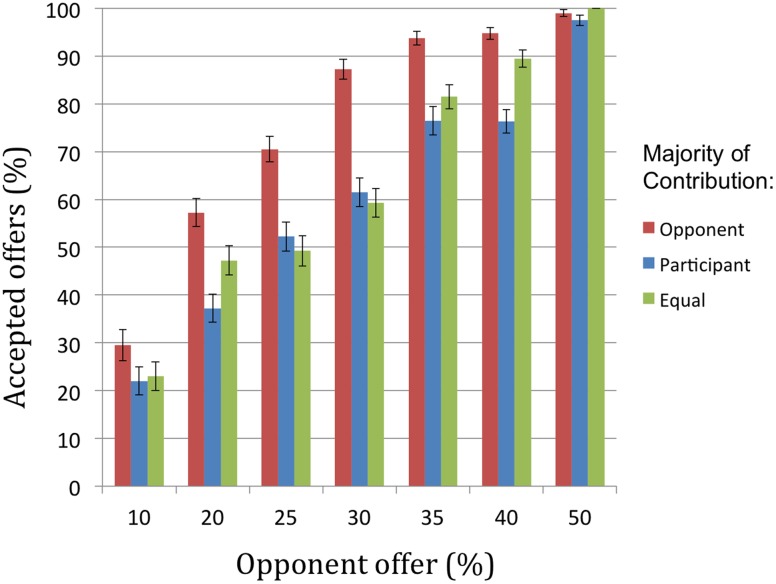
Percentage of accepted offers by opponent offer ranging from very unequal (10%) to equal (50%) for each contribution condition.

There was a significant main effect of avatar offer [*F*(3.34,665.21) = 305.37, *p* < 0.001, $\eta_{p}^{2}$ = 0.61] whereby participants accepted only 25% of the most unequal offers (10% of combined earnings) which increased to 99% of equal offers (50% of combined earnings). *Post hoc* analyses revealed that participant acceptance percentage increased monotonically as avatar offers increased, with all adjacent conditions differing significantly from each other with the exception of the contrast between 35% and 40% (see **Figure 3**).

There was also a significant main effect of player contribution [*F*(1.59,316.51) = 111.96, *p* < 0.001, $\eta_{p}^{2}$ = 0.36], with greater participant acceptance percentage when the avatar opponent contributed more compared with equal contribution trials [*t*(199) = 10.61, *p* < 0.001], and compared with trials when participants contributed more [*t*(199) = 12.06, *p* < 0.001]. Acceptance rates for equal contribution trials were significantly higher than for trials in which participants contributed more [*t*(199) = 4.79, *p* < 0.001].

Critically, we also observed a significant avatar offer × contribution interaction [*F*(9.10,1810.14) = 12.82, *p* < 0.01, $\eta_{p}^{2}$ = 0.06]. When the avatar opponent contributed more, participants accepted all unequal offers (<50%) significantly more. However, participant acceptance rates for conditions in which the participant contributed more and when contributions were equal were similar across most levels of offers, with significant differences (lower when participants contributed more) only observed at offers of 20% [*t*(199) = 4.21, *p* < 0.001] and 40% [*t*(199) = 6.56, *p* < 0.001].

To clarify this interaction further we calculated *offer sensitivity.* This measure indicates the degree to which a participant increased their inclination to accept the offer as the amount proposed by the avatar increased. Offer sensitivity was calculated in the middle of the range, using the formula: *Offer sensitivity = [2^∗^(% accepted at 40)* + *1^∗^(% accepted at 35)* -*0^∗^(% accepted at 30)* -*1^∗^(% accepted at 25)* -*2^∗^(% accepted at 20)]/overall % accepted].* Offer sensitivity was calculated separately for each contribution condition. There was a significant main effect of contribution on offer sensitivity [*F*(1.99,396.12) = 11.99, *p* < 0.01, $\eta_{p}^{2}$ = 0.06], with significant differences between each level of contribution [all *t*s(199) > 2.41, *p*s < 0.01]. Equal contributions produced the largest sensitivity (mean = 2.45, *SD* = 2.47), followed by the participant contributing more (mean = 2.06, *SD* = 2.29), and finally the opponent contributing more (mean = 1.66, *SD* = 1.87).

#### Offers Proposed

**FIGURE 4:**
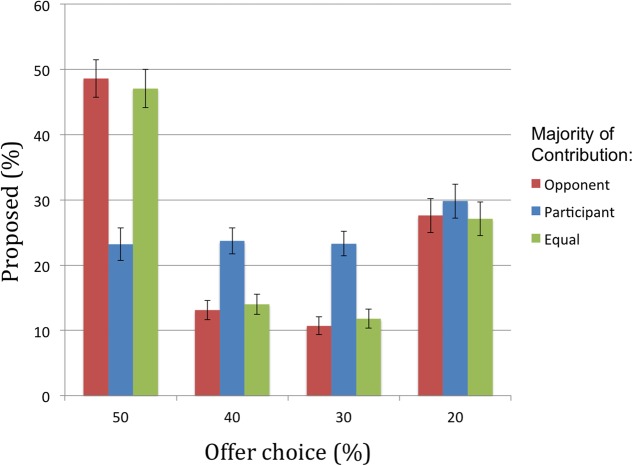
Percentage of offers proposed by participants, ranging from equal (50%) to very unequal (20%), for each contribution condition.

A main effect of participant offer [*F*(1.85, 367.76) = 25.41, *p* < 0.001, $\eta_{p}^{2}$ = 0.11] revealed there were more offers proposed at the extreme (50 and 20%) than at the intermediate levels (40 and 30%). There was also a significant contribution × participant offer interaction [*F*(4.47, 889.76) = 41.34, *p* < 0.001, $\eta_{p}^{2}$ = 0.17]. Across the different offer levels, propensity to offer did not differ significantly between conditions where the opponent contributed more and contributions were equal [*t*(199) = 0.42, *p* = 0.68]. However, when participants contributed more, we observed significantly fewer equal (50%) offers [both *t*s(199) > 10.40, *p*s < 0.001] and significantly more unequal offers of 40% [both *t*s(199) > 4.62, *p*s < 0.001] and 30% [both *t*s(199) > 6.00, *p* < 0.001]. There were no significant effects of contribution in propensity to propose highly unequal offers of 20% [all *t*s(199) < 1.85, *p*s > 0.07] (see **Figure 4**).

We observed a significant negative correlation between participant acceptance rates and generosity of proposals (*r* = -0.20, *p* = 0.005), indicating that participants who offered more tended to accept less often.

### Prisoner’s Dilemma (PD)

**FIGURE 5:**
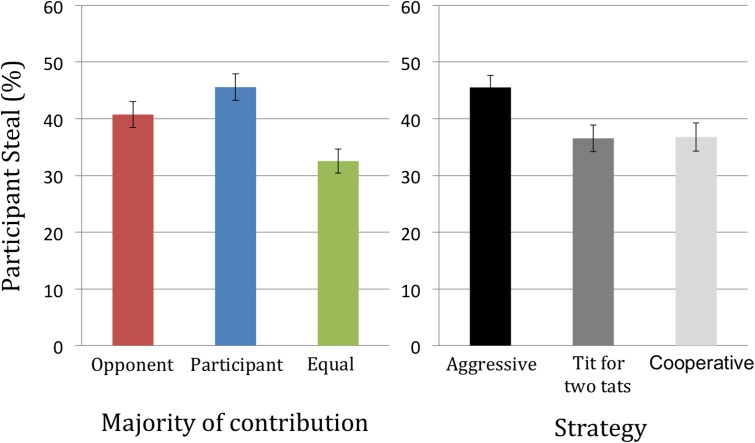
Participant defect percentage for each level of contribution and strategy.

Fourteen percent of participants cooperated consistently throughout the game, always choosing to split. Similar to the UG, there was a significant main effect of contribution [*F*(1.78,354.78) = 27.85, *p* < 0.01, $\eta_{p}^{2}$ = 0.12]. All three conditions differed significantly [all *t*s(199) > 2.47, *p*s < 0.014], with greater contributions by participants eliciting the highest defection rates, followed by greater contributions by the opponent, while defection rates were lowest when contributions were equal (see **Figure 5**).

There was also a significant main effect of avatar opponent strategy [*F*(1.87,369.57) = 12.94, *p* < 0.01, $\eta_{p}^{2}$ = 0.06], with greater defect proportions when playing against a suspicious opponent compared to a tit for two tats and a cooperative opponent [all *t*s(199) > 3.94, *p*s < 0.001]. The opponent strategy × contribution interaction was non-significant [*F*(3.79,754.69) = 0.26, *p* = 0.26, $\eta_{p}^{2}$ = 0.007)].

Since the order of contribution conditions was randomized across participants, we were able to investigate responses in the first round of play in order to explore the propensity to be cooperative from the outset. We observed that 28% of participants stole on the first round, with a significant effect of player contributions [χ^2^(2) = 7.06, *p* = 0.03]. Participants who had contributed more to the pot of money were more likely to defect on the first round (40%) compared to when the opponent contributed more (34%) and when contributions were equal (26%).

### Relationship between UG and PD

There was a significant negative correlation between PD defect percentage and the value of offers proposed in the UG (*r* = -0.48, *p* < 0.001): participants who stole more frequently in the PD offered less to their opponents on the UG. However, there was no correlation between PD defect percentage and the percentage of UG offers accepted.

### Relationship with Personality and Demographic Characteristics

**FIGURE 6:**
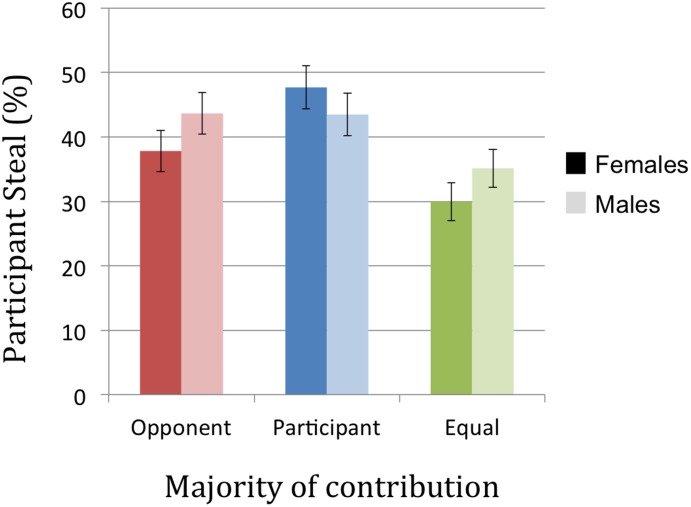
Effect of gender and contribution on PD defect rate.

There were no significant main effects of gender on participant acceptance rates [*F*(2,396) = 0.05, *p* = 0.95, $\eta_{p}^{2}$ = 0.00], offer sensitivity [*F*(2,396) = 0.03, *p* = 0.97, $\eta_{p}^{2}$ = 0.00] or offers proposed [*F*(2,396) = 1.75, *p* = 0.17, $\eta_{p}^{2}$ = 0.01] in the UG. However, in the PD we observed a significant interaction between gender and contribution on participant defect rates [*F*(2,396) = 5.17, *p* < 0.05, $\eta_{p}^{2}$ = 0.03]. Females’ defect behavior differed significantly between each level of contribution [all *t*s(199) > 3.1, all *p*s < 0.001], effectively driving this main effect described above. Equal contributions elicited the lowest defect rates, with the highest defect rates when participants contributed more. Males, however did not vary their behavior significantly between conditions where they contributed more compared to when the opponent contributed more [*t*(199) = 0.08, *p* = 0.94], though defect rates in both of these conditions were significantly higher than equal contributions [both *t*s(199) > 3.70, *p*s < 0.001] (see **Figure 6**).

Overall acceptance rates in the UG correlated positively with state anxiety (STAI State: *r* = 0.26, *p* < 0.001; POMS tension-anxiety: *r* = 0.24, *p* < 0.001) suggesting that people experiencing higher levels of negative affect were more likely accept their opponent’s offer.

## Discussion

Economic social exchange games are widely used to study cooperative behavior, yet two central caveats of these games is that first, players must divide an arbitrary sum of money to which they have not contributed and therefore may have little investment in. However, rarely in real life is bargaining behavior and cooperation devoid of motivating factors such as investment and contribution. In this study we found that contribution to the monetary stake robustly affects the propensity to engage in cooperative behavior. Second, economic games used to study social cognitive decision-making typically use a complex set-up of pairing participants, which is not practical. Here we observed behavior similar to previously reported real-time UG and PD therefore suggesting that games using computer avatars may be an effective alternative to a traditional set up.

As expected, we found that UG acceptance rates increased as avatar opponent offers become fairer (e.g., Güth et al., 1982). As we hypothesized, contribution had a substantial effect upon both acceptance rates and proposals. The highest acceptance rates were evident consistently when the avatar opponent contributed more, suggesting that people are more likely to consider an unequal offer as fair when they have made a lower contribution. Consistent with this, when participants acted as proposers, they made fewer 50% offers when their own contribution was greater, again indicating that relative contribution impacts perceptions of fairness. We also observed that when playing the role of the proposer, participants made more offers at the extremes, i.e., equal (50%) and very unequal offers (20%) compared with intermediate offers (40 and 30%). This is in line with the results of meta-analyses, suggesting that dictators’ offers in the DG follow a bimodal distribution, with a high concentration of purely selfish and perfectly fair offers (Engel, 2011; Tisserand et al., 2015). This suggests that perhaps the participants played more like a dictator against a passive opponent as opposed to a typical proposer in the ultimatum game that must have strategic considerations in order to avoid the rejection of the offer. It might therefore be interesting in future studies to manipulate an avatars acceptance threshold in order to ascertain how it might affect players’ proposer behavior.

We observed a small (*r* ∼-0.2), but significant, negative correlation between UG receiver and proposer behavior whereby participants who offered more also accepted fewer offers. This replicates previous findings suggesting that a participant with a high minimum acceptable offer will expect others to behave similarly and therefore increase their offer (Blanco et al., 2011). Additionally we detected a moderate (*r* ∼-0.5) negative correlation between defect rates in the PD and offers proposed in the UG in line with previous DG studies (Dreber et al., 2013; Capraro et al., 2014). However, despite good statistical power, we did not identify a significant correlation between PD and UG offer acceptance rates, suggesting that these games may measure dissimilar underlying processes. Previous studies have also observed a lack of correlation between UG acceptance and PD cooperation, which has been attributed to the PD reflecting cooperation and reciprocity, with the UG acceptance rates reflecting assertiveness and a tendency to avoid being dominated by the proposer (Yamagishi et al., 2012).

We further demonstrated that acceptance rates in the UG correlated positively with state anxiety, suggesting that negative affect is an important factor in cooperative behavior. Previous studies have reported that anxious traits lead to greater cooperation (Clark et al., 2013) and individuals with anxiety and depressive disorders are more likely to display cooperative behavior (McClure et al., 2007) and accept significantly more unfair offers than healthy controls (Grecucci et al., 2013).

As expected, our PD results showed that the highest defect rates occur when healthy volunteer participants contributed more to the financial pot, suggesting that they feel entitled to a larger share of the pot in this situation. However, defect rates were also higher when the computer opponent contributed more compared to when contributions were equal, which seems counterintuitive. A possible explanation is that participants may predict that their opponent is more likely to defect in this scenario, and therefore may also choose to defect in order to sabotage their opponent’s gains (i.e., in this case participants would rather see both players receive nothing than allow their opponent to take all the money; a form of punishment behavior). Previous studies have demonstrated that participants’ prediction of cooperation is extremely accurate (Frank et al., 1993; Brosig, 2002) such that they are inclined to play cooperatively against those who are expected to cooperate and to defect against those more likely to defect (Oberholzer-Gee et al., 2010).

We also observed that whilst overall cooperative behavior does not differ between males and females, the manipulation of contribution produces clear gender differences. Specifically, females defect more frequently when they have contributed more, whereas males’ defecting behavior is also relatively high when the opponent has contributed more. The defect response to predicted opponent defection hypothesized in the preceding paragraph would therefore seem to be particularly marked in male participants, potentially consistent with the idea that males are more prone to “altruistic punishment” of perceived unfairness (Singer et al., 2006). Whilst previous literature regarding gender differences in the PD is mixed (Rapoport and Chammah, 1965; Sibley et al., 1968; Tedeschi et al., 1969; Kahn et al., 1971; Mack et al., 1971; Dawes et al., 1977; Orbell et al., 1994), here we demonstrate the importance of considering contribution as a key factor influencing cooperative behavior between the sexes. Interestingly, the genders did not differ on our task when contributions were equal, most similar to typical implementations of the PD.

Many studies using the UG and PD employ time consuming and elaborate set-ups. However, we found that despite participants being fully aware that they were playing against a computer opponent, the degree of cooperative behavior when both players contributed equally was comparable to previous studies (Güth et al., 1982; Rubinstein, 1982; Thaler, 1988). However, some studies have found higher rejection rates for human compared to computer avatar opponents (Blount, 1995; Knoch et al., 2006). In the PD our rates of cooperation of 60% were slightly greater than the average figure of 47% obtained in a meta-analysis of 130 single shot PD experiments (Sally, 1995). Nevertheless, more recent real life studies also show slightly greater acceptance rates: van den Assem et al. (2011) analyzed 574 contestants’ behavior from the United Kingdom game show “Golden Balls,” adapted from the Prisoner’s Dilemma, finding that players cooperate on average 53% of the time. Moreover, during piloting of the tasks we found no significant differences between games played against the computer opponent and games played against a confederate human opponent. Therefore computer avatars may serve as a suitable and more practical alternative to the traditional setup. Indeed, it has been argued that human–computer interactions are evolving with computers increasingly being viewed as more human-like with autonomous interactions (Cassell and Bickmore, 2000). Remarkably, the substantial effects of contribution suggest that participants treat the computer avatar as though they have exerted effort much like a human opponent. It should be noted that we recruited relatively young participants who are more likely to be familiar with human–computer interactions such as gaming and the use of humanized computer avatars. We may have seen a different pattern in an older population.

A limitation of our findings is that money was accrued differently in the two games; “won” in the UG and “earned” in the PD. Recent experimental work on social preferences has shown that people are much more willing to accept inequalities due to effort than inequalities due to luck (Cappelen et al., 2007, 2013; Konow, 2010). However, in neither game was the pot of money a “free gift” from the experimenter. Evidence suggests that people who believe economic outcomes mainly depend on effort show less cooperative behavior whereas those who believe that other factors not under an individual’s control determine economic outcomes engage in more social cooperative behaviors such as wealth redistribution and charitable donations (Rey-Biel et al., 2011). Indeed, entitlements represent a strong motive over above consideration for needs and nationality that may contribute to explaining why people do not give a larger share of income to the more needy (Cappelen et al., 2013). In addition, potential framing of the tasks, which used phrases such as “win” and “steal,” is an important caveat that has been shown to influence cooperative behavior (e.g., Ellingsen et al., 2012). An interesting future direction would be to manipulate the framing of the tasks to ascertain how it affects cooperative behavior particularly in a human–computer interaction.

Another possible limitation of the study is that the offers in the UG had seven levels: 10, 20, 25, 30, 35, 40, or 50% whereas when players take the role of dictator they only have four offer options; 20, 30, 40, or 50%. In future versions of the tasks it would be valuable to match the offer levels in the two roles and to perhaps consider including opponent offers above 50%. For potential further studies, it would be interesting to evaluate whether behavior differs if the avatar of the computer opponent is replaced by an image of a computer screen, or a neutral stimulus such as a gray circle. This would allow investigation into the effect of using human-like avatars in these economic games. Further systematic exploration assessing human–human and human–computer interaction is needed to establish whether cooperation with an avatar provides real insight as to an individual’s cooperation with other human agents.

Finally, an important limitation of this study is that we have only used two games, which by no means comprehensively assess cooperative behavior. The PD and UG are very different games and are likely to be manipulating different aspects of behavior. Furthermore, in the versions we used here, the method of accruing endowments were not matched and the UG was played as a one shot game while the PD was repeated. Future studies should employ additional tasks and systematically manipulate game variables across these tasks in order to more fully characterize the behavior we report here.

## Conclusion

Taken together, our results demonstrate that contribution is an important motivating factor that affects behavior in economic social exchange games. Traditional versions of these games involve the experimenter providing an arbitrary pot of money that must be divided. In the versions used here, participants either won or earned money, making either equal or unequal contributions to the communal pot. This version of the UG allowed us to show that perceptions of fairness depend on how potential rewards have been accrued. Participants were more accepting of unequal offers when they had made a smaller contribution, and less likely to make equal offers when they had made a larger contribution. For the PD we found that participants were more likely to split earnings when contributions were equal and females were more likely to defect when they had contributed more. Males were more likely to defect either when they had contributed more or when the opponent had and we suggest that the latter behavior may represent anticipatory punishment of a predicted defect by the opponent. Our findings have important implications for understanding perceptions of fairness and cooperative behavior in real-life situations where people have expended effort together to achieve gains that must then be divided.

## Author Contributions

Chief Investigator: RE. Principal Investigators: JR, MM, BS, and TR. AB and TS collected and analyzed data.

## Conflict of Interest Statement

The authors declare that the research was conducted in the absence of any commercial or financial relationships that could be construed as a potential conflict of interest.
